# Screw fixation in stemless shoulder arthroplasty for the treatment of primary osteoarthritis leads to less osteolysis when compared to impaction fixation

**DOI:** 10.1186/s12891-020-03277-3

**Published:** 2020-05-12

**Authors:** Arad Alikhah, Jan-Phillipp Imiolczyk, Anna Krukenberg, Markus Scheibel

**Affiliations:** 1 Department of Shoulder and Elbow Surgery, Center for Musculoskeletal Surgery, Charité-Universitaetsmedizin Berlin, Augustenburger Platz 1, 13353 Berlin, Germany; 2 Department of Shoulder and Elbow Surgery, Schulthess Clinic, Zuerich, Switzerland

**Keywords:** Stemless shoulder arthroplasty, Primary osteoarthritis, Screw fixation, Impaction design

## Abstract

**Background:**

Stemless total shoulder arthroplasty is a well-established and reliable surgical treatment option for glenohumeral osteoarthritis resulting in loss of pain and improvement of shoulder function. Currently the two methods for the fixation of the humeral component are either screw fixation or impaction. The purpose of this study is the clinical and radiological comparison of two different stemless designs (screw fixation vs impaction) for total shoulder arthroplasties in patients suffering from primary glenohumeral osteoarthritis.

**Methods:**

A retrospective cohort study including 39 patients with a mean age of 67 years and a minimum follow-up of 2 years was performed. Patients were separated into two groups based on the selected implant. In group A (*n* = 18) a screw fixation design and in group B (*n* = 21) an impaction type design was used. For clinical examination the Constant-Murley-Score (CS) and Subjective-Shoulder-Value (SSV) were evaluated. Radiological examination was performed on true-AP, axial and Y-view radiographs.

**Results:**

In group A the CS increased from 27.1 to 65.2 points and SSV from 27.3 to 76.7% (*p* > 0.05). No osteolysis of the medial calcar or subsidence of the humeral implant were found in this group. In group B the CS increased from 29.0 to 72.6 points and SSV from 33.1 to 85% (*p* < 0.05). Osteolysis of the medial calcar was present in seven patients in this group. No signs for humeral loosening were found in both groups.

**Conclusion:**

Impaction and screw fixation total shoulder arthroplasty for primary glenohumeral osteoarthritis using a stemless device provide reliable clinical results. The screw fixation seems to prevent osteolysis of the medial calcar.

## Background

Total shoulder arthroplasty is a well-established and reliable surgical treatment option for glenohumeral osteoarthritis resulting in loss of pain and improvement of shoulder function [1]. Due to excellent clinical results usage and therefore also revisions become more frequent [2–4]. Stemless shoulder replacement was introduced to shorten operation time, reduce stemmed related complications, save bone stock and ultimately make revisions easier [5–7]. Stemless endoprosthesis are implanted through humeral anchoring in the epiphyseal and/or metaphyseal bone and achieve a canal sparing fixation [8, 9]. A number of studies have shown radiological reliability and clinical improvements that are comparable with stemmed designs in short and midterm follow-up [6, 10–12]. Currently there are two methods of humeral fixation. Designs use either an impaction method for anchoring the humeral component or a hollow screw [9, 13, 14].

The purpose of this study is the clinical and radiological comparison of two different stemless design (impaction vs. screw fixation) of stemless total shoulder arthroplasties for patients with primary glenohumeral osteoarthritis. Our null hypothesis was that there is no difference in clinical and radiological results between the two implants.

## Methods

A retrospective cohort comparison including patients with primary osteoarthritis of the glenohumeral joint was used for this study. This study was approved by the ethics committee of our institution (EA2/154/18). Written informed consent was obtained from all patients enrolled in this study. Inclusion and exclusion criteria are listed in Table 1. Of the 39 included patients 11 were male and 28 were female. Mean age was 67 (44–83 median: 69) years. All patients were evaluated clinically and radiologically with a standardised protocol with a minimum of 2 years follow-up. Clinical outcomes were documented using the Constant-and-Murley Score (CS) [15]. The overall satisfaction was evaluated using the Subjective-Shoulder-Value (SSV). Standard radiographs (true anterior-posterior, axial and Y-views) were taken to evaluate for signs of loosening or osteolysis according to previous studies [6, 16, 17]. For the evaluation of glenoid loosening the Molé-Score was used [16]. For the radiological evaluation we always compared the 6 weeks postoperative and the last follow-up radiographs. Additionally, intra- and postoperative complications were documented. Patients received the modell of endoprothesis based on which year they were treated. Initially we used a screw fixation method in our clinic, later we switched to an impaction method.

**Table 1:** Patients inclusion and exclusion criteria for this study

Inclusion criteria	Exclusion criteria
Patients are at least 18 years old / Skelettaly mature	Any other shoulder related pathologies besides primary osteoarthritis of the glenohumeral joint
The patient is willing and able to cooperate with the required postoperative therapy	Disagrees with participation in the study
Was diagnosed with primary osteoarthritis of the glenohumeral joint and required total shoulder arthroplasty	
Complete clinical and radiographic examinations at each required appointment	
Minimal Follow-up of 24 months	

### Surgical procedure

All surgeries were performed by the same senior surgeon. Patients underwent general anaesthesia combined with an interscalene block for optimal pain relief. All patients were placed in beach-chair position. A deltopectoral approach and a subscapularis tenotomy was used in all procedures. A capsular release and a tenotomy of the long head of the biceps tendon was performed and the humeral osteophytes were removed. Implantation of the endoprosthesis was performed following the manufactures description. Two different models of stemless shoulder arthroplasties were implanted.

**Fig. 1:**
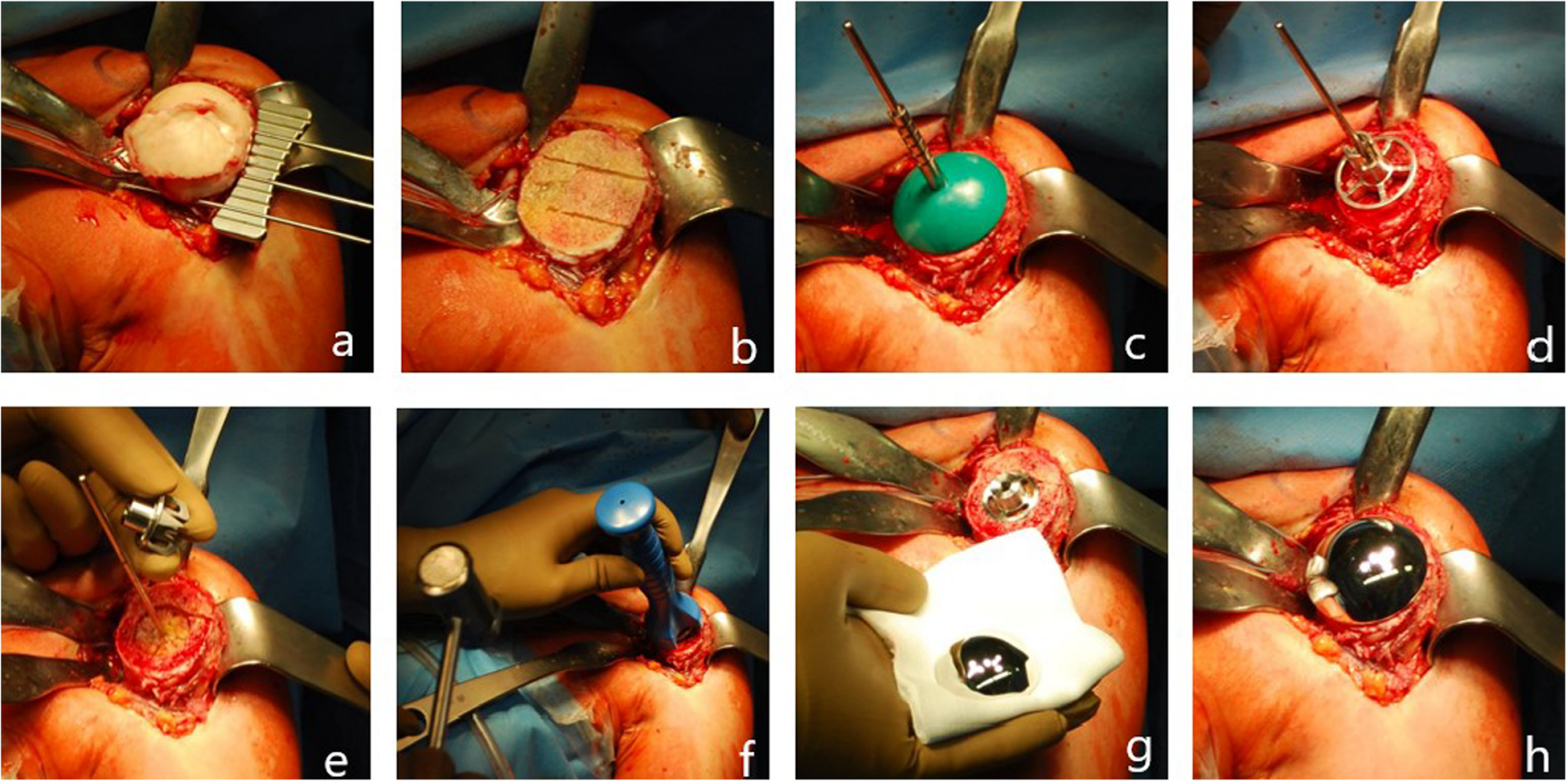
**a**–**c** Surgical technique of the Eclipse Shoulder Prothesis

In the study group (group A) (*n* = 18) the Eclipse Shoulder Prosthesis (Arthrex, Inc., Naples, FL, USA) was used (Fig. 1a–c). This system consists of three humeral components. A central hollow screw sometimes referred to as a cage that fixates a baseplate also called trunion to the anatomical neck. The third component is the humeral head. There are different resection guides corresponding to different sizes of humeral heads. The retroversion was determined according to the patient’s anatomic neck and the guide attached with a Steinmann pin leading the way for two K-wires in the proximal humerus. Once the K-wires were placed the resection guide was removed and the humeral head resected at the anatomic neck. Using templates, the trunion size was determined. After preparation of hole for the cage screw using a reamer a protection plate was placed on the resection area during replacement of the glenoid. Afterwards a cage screw sizer was drilled through a centering device until it reaches the lateral cortex. This process determined the length of the screw. Drill template and cage screw sizer were removed and the baseplate was fixed over the centering devise using an impactor. The cage screw is screwed in over the trunion while pressing the trunion to the resection area. The appropriate head was determined with the help of trial heads. Finally the humeral head is impacted on the trunion.

**Fig. 2:**
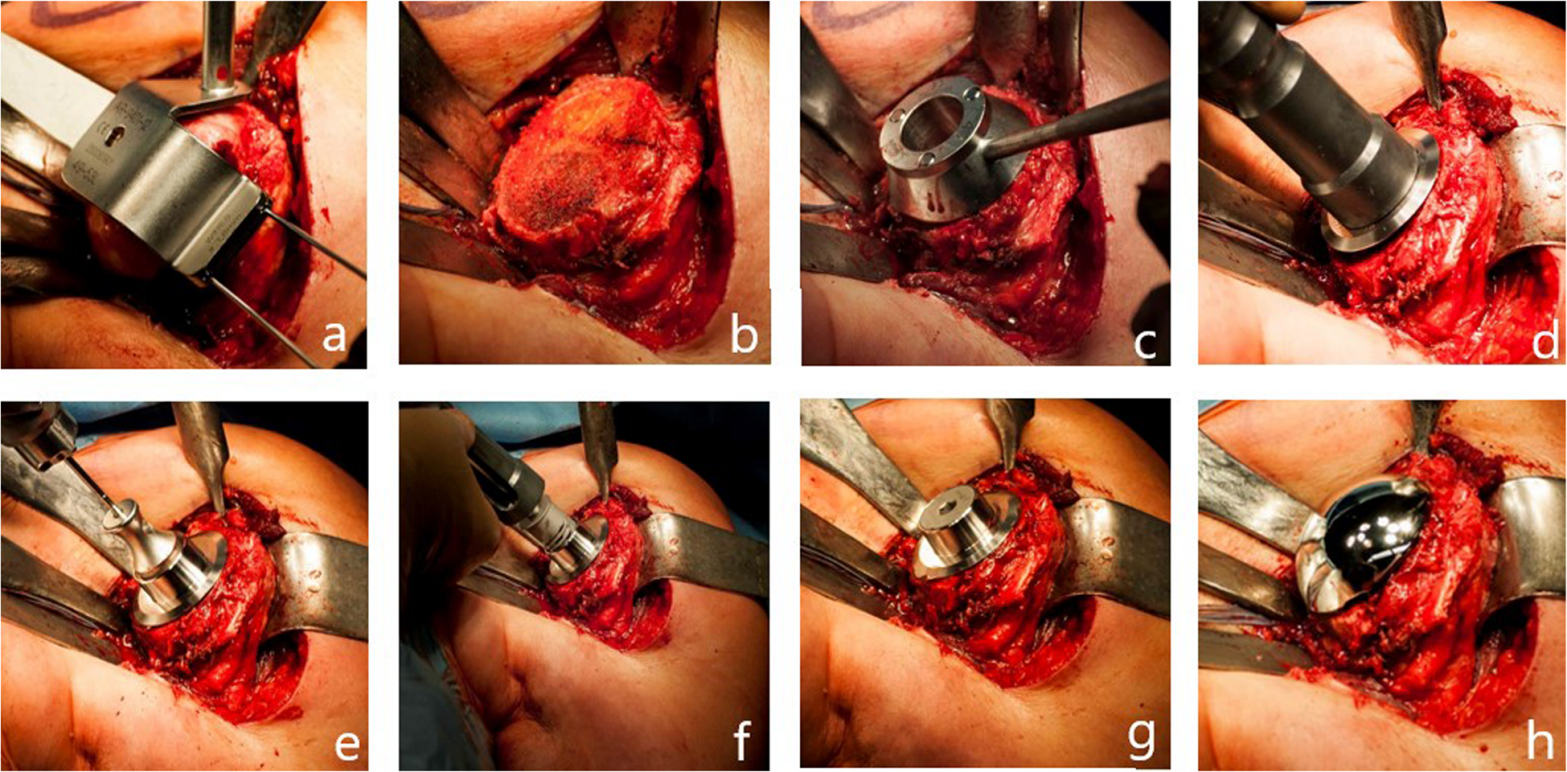
**a**–**c** Surgical technique of Sidus Stem-Free Shoulder System

In the control group (group B) (*n* = 21) the Sidus Stem-free Shoulder System (Zimmer Biomet, Inc., Warsaw, Indiana, USA) was used (Fig. 2a–c). This system is comprised of two parts. A grit blasted titanium anchor and a cobalt chrome humeral head. For resection of the humeral head a guide was used. This guide was positioned at the medial border of the insertion of the supraspinatus tendon using two K-wires. The inclination angle and retroversion were determined with the help of this resection guide. After resection at the anatomic neck a protection plate was placed on the resection plane during preparation of the glenoid. Once the glenoid was replaced a central pin was placed through a trial head. This pin was used for preparation of the humeral fixation side. After a slightly undersized preparation of the humeral bone with a drill and a puncher the anchor was impacted into the humeral head through the same pin. Three different sizes of anchors are available. After testing with a trial head, the final cobalt chrome head is placed on the anchor. The anchor and head are connected by a Morse taper connection.

Both systems were combined with all polyethylene fully cemented keel glenoid. The labrum was resected and the capsule was released around the glenoid and the glenoid was prepared using a reamer and a keel punch. The size of the glenoid was determined with the help of trial glenoids. The definite glenoid was implanted with a high-pressure cement application into the bone and application on the backside of the polyethylene keel glenoid including the keel. The glenoid component was then impacted into the bone. After all arthroplasties were implanted subscapularis repair and wound closure were performed in a standard fashion.

### Postoperative rehabilitation

Postoperatively patients followed a standardized rehabilitation protocol. The shoulder was immobilised in a sling in internal rotation for 6 weeks. During this time, only passive movement (excluding external rotation) above zero degrees was allowed during physiotherapy sessions. Movement was slowly increased under supervision of the physiotherapist. After 6 weeks active motion was added to the protocol. Strength exercises were carefully introduced after full range of motion was achieved.

### Data collection & analysis

Patients were examined by two parties and the functional scores were documented on datasheets. The radiographs were anonymously evaluated. Data were then statistically analyzed using Excel (Microsoft office 2016, Microsoft cooperation, Redmond, Washington, USA). We used a two sample t-test to determine significance. Because we presumed that both sets of data were independent and had the same potential for variance. The level of significance was set at below 0.05.

## Results

### Demographic results

Mean age was 67 (range: 44–83 years median: 69) years at the time of operation. Average follow-up was 36.3 (24–72) months. Demographic data are summarized in Table 2. Of the 18 patients in group A, five were male and 13 were female. Mean age was 66.7 years (range: 44–81 years median: 69). Average follow-up was 42.2 (24–72) months. Of the 21 patients in group B, six were male and 15 were female. Mean age was 67.4 years (range: 55–83 years median: 71).

**Table 2:** Compared demographic data between both groups

	Group A	Group B	*p* value
*N*	18	21	
Mean age in years	66.7 (44–81)	67.4 (55–83)	0.835
Mean follow-up in months	42.2	36.3	0.010

The average follow-up for group B was 30.3 (24–48) months.

### Functional results

Patients in the group A revealed an increase in CS from 27.1 to 65.2 points (*p* < 0.001). The subcategories increased from 6.4 points to 13.4 points in pain, 8 points to 17.4 in activities of daily living, 11 points to 26.8 points in active range of motion and from 1.6 points to 7.2 points in strength (*p* < 0.001). The SSV increased from 27.3 to 76.7% (p < 0.001).

Preoperatively in group B there were 14 type A glenoids and 7 type B glenoids according to Walch et al. Patients in group B revealed an increase in CS from 29.0 to 72.6 points (*p* < 0.001). In the specific subcategories an increase from 6.2 points to 13.7 points in pain, 8.5 points to 19.2 points in activities of daily living, 13 points to 33.2 in active range of motion and from 0.93 points to 7.3 points in strength was observed (p < 0.001). The average SSV here increased from 33.1 to 85% (*p* < 0.001).

**Fig. 3:**
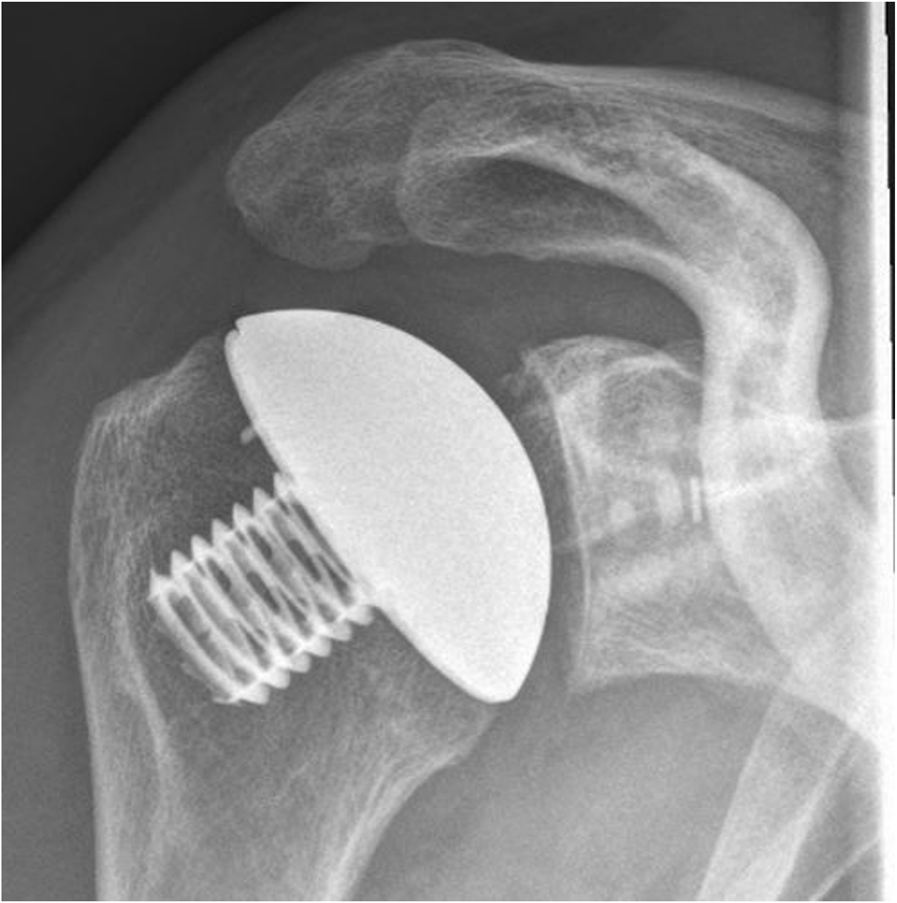
Radiograph shoulder in true-AP: eclipse without calcar resorption

**Fig. 4:**
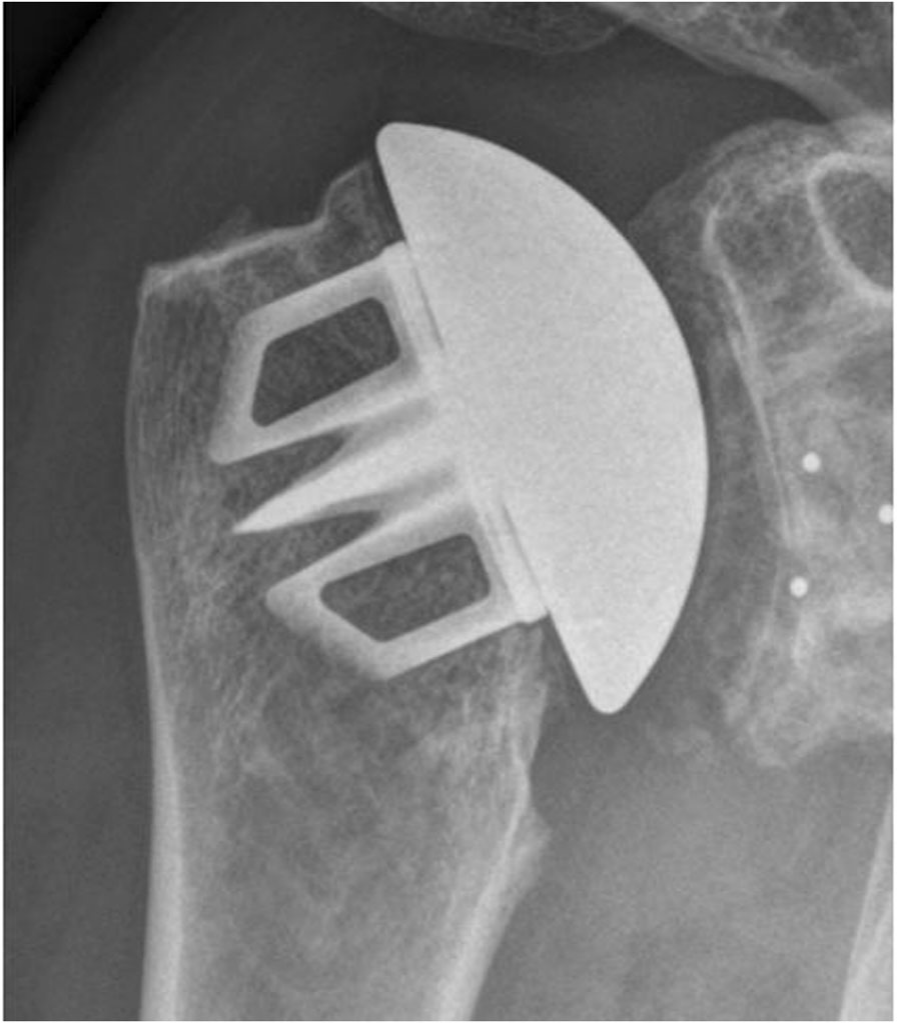
Radiograph shoulder in true-AP: Sidus with calcar resorption

There was no significant difference in CS and SSV between the groups (*p* = 0.167). Functional results are summarized in Table 3 and Figs. 3 and 4.

**Table 3:** Preoperative and postoperative clinical results of both groups

	Group A	Group B	*p* value
Constant- Murley-Score (preoperative)	27.1 points	29 points	0.762
Constant-Murley-Score (last Follow-up)	65.2 points	72.6 points	0.167
Subjective-Shoulder-Value (preoperative)	27.3%	33.1%	0.432
Subjective-Shoulder-Value (last Follow-up)	76.7%	85%	0.378

### Radiological results

Preoperatively in group A there were 13 type A glenoids and 5 type B glenoids according to Walch et al. [18].

**Fig. 5:**
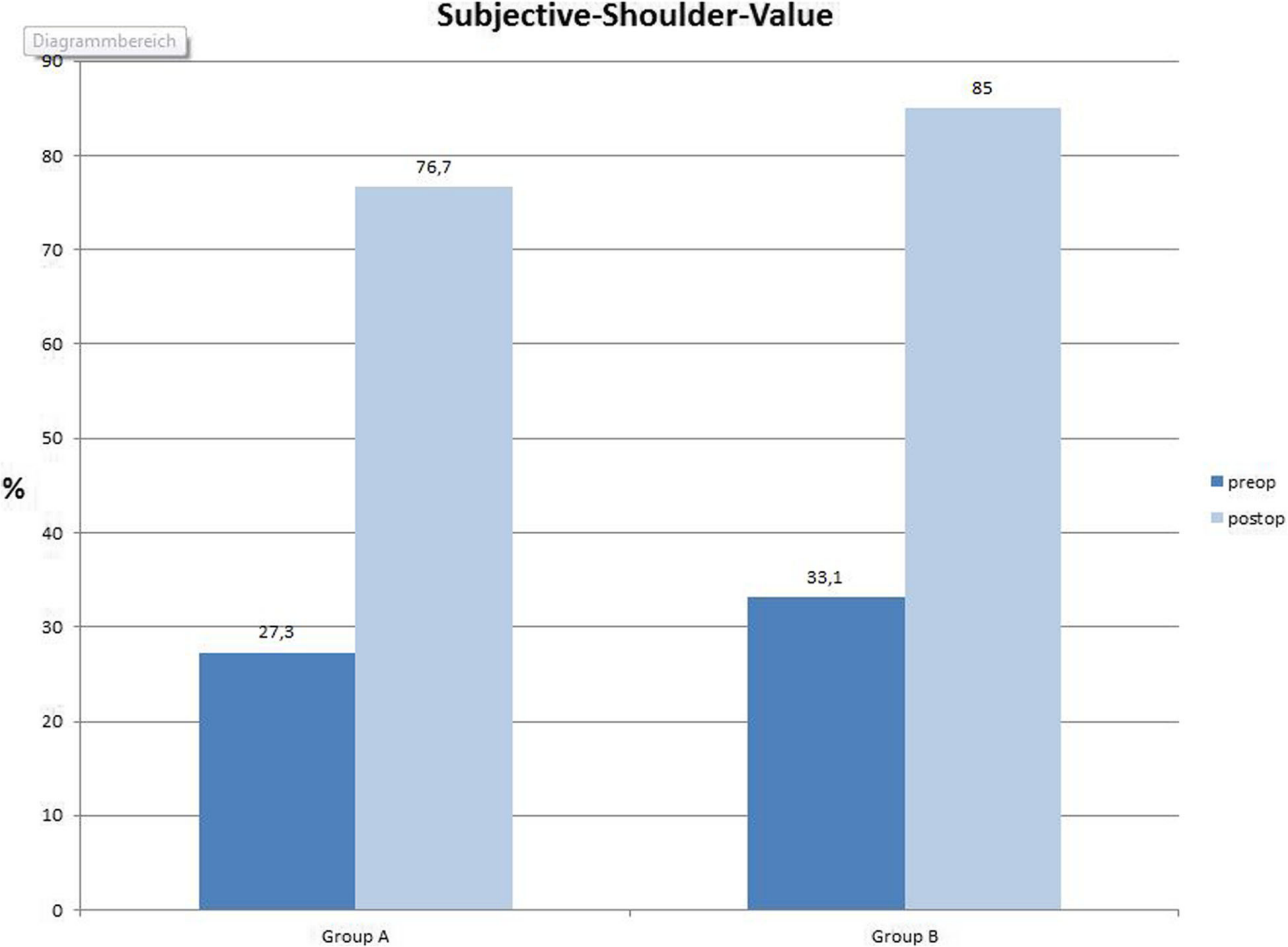
Clinical results Subjective-Shoulder-Value

In group A we found radiolucent lines as signs of glenoid loosening in five patients (Mole-Score = 0.25). There were neither signs of humeral loosening, osteolysis nor osteophytic exostosis (Fig. 5).

**Fig. 6:**
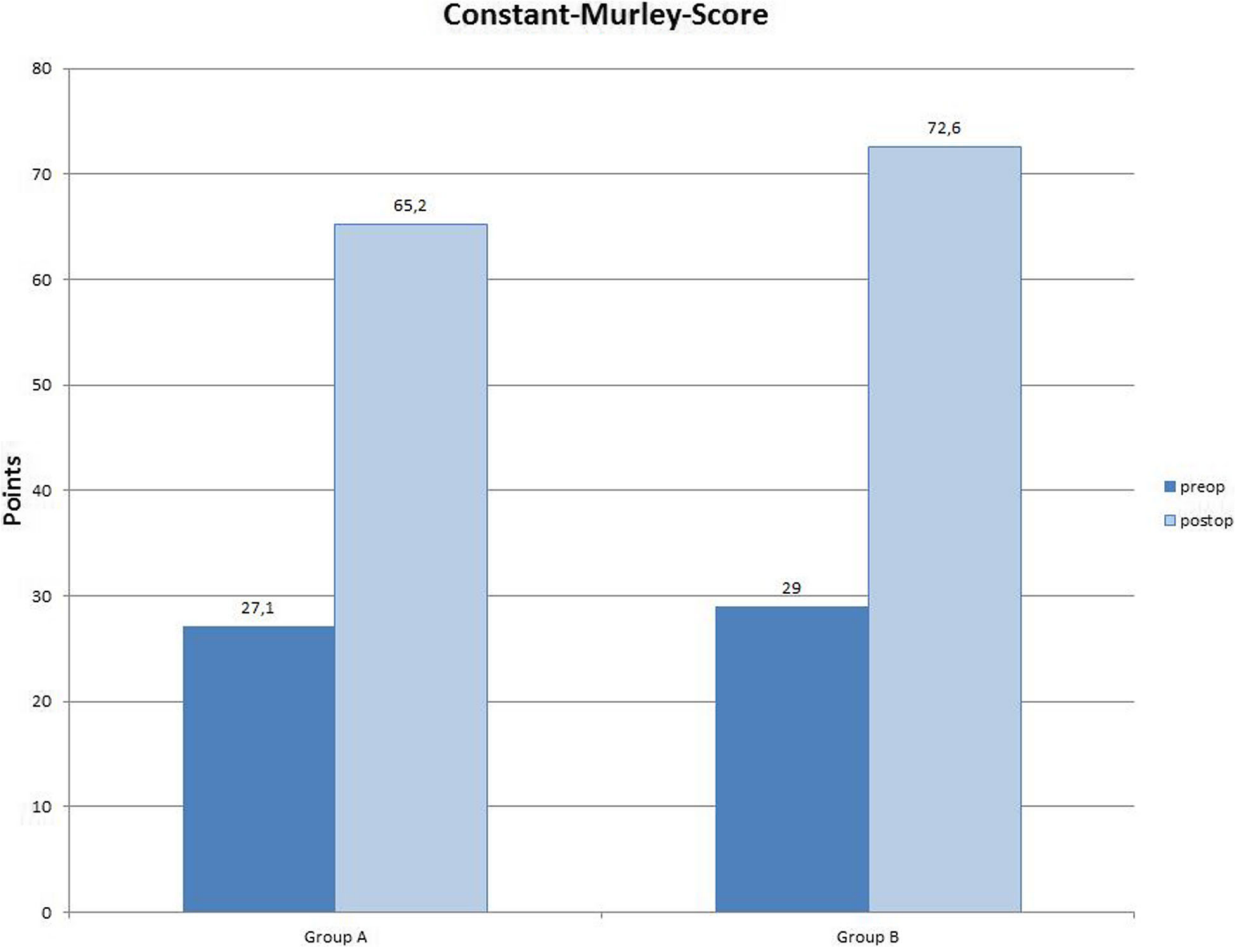
Clinical results Constant-Murley-Score

In group B four patients presented mild signs for glenoid loosening (Mole-Score = 0.89). We found osteolysis of the medial calcar in seven patients (Fig. 6). Humeral loosening and osteophytic exostosis were not found.

There were no significant differences in loosening and osteophytic exostosis between the groups (*p* = 0,68). We found significantly more osteolysis in group B (*p* < 0.003). Radiographic results are summarized in Tables 4 and 5.

**Table 4:** Preoperative and postoperative Constant-Murley-Score subcategory of both groups

	Group A		Group B		
	Preoperative	Last follow-up	Preoperative	Last follow-up	
Pain	6.4 points	13.4 points	6.2 points	13.7 points	0.147
Everyday activity	8 points	17.4 points	8.5 points	19.2 points	0.499
Range of motion	11 points	26.8 points	13 points	33.2 points	0.272
Strength	1.6 points	7.2 points	0.93 points	7.3 points	0.144

**Table 5:** Postoperative radiologic results of both groups

	Group A	Group B	*p* value
Loosening (humeral)	*N* = 0	*N* = 0	0.500
Loosening (glenoidal)	Mole score = 0.25	Mole score = 0.89	0.269
Osteophytic exostosis (humeral)	*N* = 0	*N* = 0	0.500
Calcar resorption	*N* = 0	*N* = 7	0.005

### Complications

We examined the electronical medical records and scanned for adverse events. We found complications in two patients. Complication rate was therefore 5.1%. Revision rate in this cohort was 2.6%. During anchor placement an intraoperative fracture at the greater tuberosity was recorded in group B. With no dislocation no further treatment was initiated and radiologically the fracture healed well. In group A a suspected low-grade infection with glenoid component loosening was treated with arthroscopic removal of the glenoid combined with the expiration of samples for microbiological examination. In a second step the endoprosthesis was revised to a reverse shoulder arthroplasty.

## Discussion

By now several studies showing the benefits of stemless total shoulder arthroplasty have been published [2, 6, 10, 13, 17, 19]. Additionally, a finite element analysis concluded that stem length reduction resulted in a more physiological stress distribution on the humeral bone [20]. Results seem encouraging and are similar to other surgeon’s experiences. Our null hypothesis (H0) turned out to be only partially true. Both designs evaluated in this study revealed a significant increase in function and patient satisfaction and decrease in pain levels. Improved clinical results were shown with both designs and radiologically both model’s results are satisfying. But osteolysis of the medial calcar only appeared in the impaction design group. This might be the result of an uneven load distribution on the humeral bone. The Eclipse shoulder prosthesis might have a better distribution because of the screw fixation design in combination with the baseplate. The baseplate might distribute the load evenly leading to constant rim loading resulting in less bony resorption. The pressure in the Sidus Stem-free Shoulder System is conducted through the anchor and from there to bone.

Another explanation for this difference in radiological results is a biological reaction to a polyethylene wear of the glenoid component or impingement of the implant against the medial calcar (humeral notching). We used fully cemented keeled glenoids from each company with both designs. One explanation could be a different humeral-glenoid mismatch comparing both designs, however this remains a subject for further studies.

There is some evidence that radiological changes do not influence clinical results in short- and mid-term follow-up [21].

This study has some limitations. The number of patients in each group is relatively small and the study has a retrospective design. Neither the patients nor the examiners were blinded and the comparison was only based on short to mid-term results. We did not quantify bone quality as a preoperative mesurement. We used the thumbtest as described by Churchill et al. to evaluate all patients [11]. Only if the test was negative we implanted one of the two endoprothesis. After switching to an impaction method there might be a learning curve for the surgeon which can lead to difference in result. Furthermore, the method of examining radiolucent lines commonly used as indication for loosening seems to have limitations of its own [21, 22].

The strength of this study lies in the uniformity of patient treatment and examination. All patients were diagnosed and treated by the same surgeon and all data were collected by the same examiner who was not the operating surgeon. Even though this evidence is not conclusive it is promising and worthy of further observation in follow-up. A finite element analysis might also be useful to explain our results.

## Conclusion

After a minimum of 24 months follow-up satisfying results can be achieved using either an impaction or a screw fixation stemless total shoulder arthroplasty. Clinical results in short-term follow-up do not differ from each other. The screw fixation seems to prevent osteolysis of the medial calcar.

## Data Availability

The datasets used and/or analysed during the current study are available from the corresponding author on reasonable request.

## Abbreviations

vs: Versus
n: Number
CS: Constant-Murley-Score
SSV: Subjective-Shoulder-Value
