# Correction to: Screw fixation in stemless shoulder arthroplasty for the treatment of primary osteoarthritis leads to less osteolysis when compared to impaction fixation

**DOI:** 10.1186/s12891-020-03360-9

**Published:** 2020-06-06

**Authors:** Arad Alikhah, Jan-Phillipp Imiolczyk, Anna Krukenberg, Markus Scheibel

**Affiliations:** 1 Department of Shoulder and Elbow Surgery, Center for Musculoskeletal Surgery, Charité-Universitaetsmedizin Berlin, Augustenburger Platz 1, 13353 Berlin, Germany; 2 Department of Shoulder and Elbow Surgery, Schulthess Clinic, Zuerich, Switzerland

**Correction to: BMC Musculoskelet Disord 21, 295 (2020)**

**https://doi.org/10.1186/s12891-020-03277-3**

Following publication of the original article [1], the authors reported the following errors found in the main text.

i) The subpanels found in the legend of Figs. 1 and 2 should be read as “**a-h**” instead of “**a-c**”.
ii) “Prothesis” is incorrectly spelt in the legend of Fig. 1. It must be read as “Prosthesis”.
iii) The images of Figs. 1 and 2 must be swapped (but not the legends).

The original article [1] has been updated.

## References

[1] Alikhah A, Imiolczyk J, Krukenberg A (2020). Screw fixation in stemless shoulder arthroplasty for the treatment of primary osteoarthritis leads to less osteolysis when compared to impaction fixation. BMC Musculoskelet Disord.

